# Book Review

**Published:** 1948

**Authors:** 


### Reviews of Books

**Medical Annual, 1947.** Edited by Sir Henry Tidy, K.B.E., M.D., F.R.C.P., and A. Rendle Short, M.D., B.S., B.Sc., F.R.C.S. Pp. 464. Illustrated. Bristol: John Wright & Sons Ltd. 1947. Price 25s.—This work has been previously referred to as the " favourite compendium of the English-speaking medical world," and the present edition is indeed a worthy successor to all that has gone before. Helpful articles and excerpts on every branch of medicine and surgery are written clearly and succinctly by recognized authorities. We can mention only a few items from its wide range: prostatectomy; pulmonary embolism; testing the viability of gut by fluorescin; tracer substances; vital statistics; vitamins and nutrition; prophylactic immunization against phthisis; modern treatment of thyroid dysfunction; epidemic thrombo-phlebitis; penicillin in syphilis; streptomycin; acute rheumatism in children; infantile paralysis; disc herniations; the surgical treatment of hyper-tension; histamine antagonists; the coronary thrombosis; the rhesus factor; and many other equally interesting subjects. The reader has only to dip into this book to realize that it is a masterly exposition of modern medicine and surgery, and that the *Medical Annual* has in fact become an institution in itself.

**Sex Fulfilment in Married Women.** By Helena Wright, M.B., B.S. Pp. 96. London: Williams & Norgate Ltd. 1947. Price 5s.—This little book is a sequel to the author's *Sex Factor in Marriage* after sixteen years of further experience. It is a melancholy fact that, although completely happy and harmonious marriage is very difficult without full physical satisfaction of both partners, many if not most women never experience such complete satisfaction or are even aware of what an orgasm is. This is largely due to tradition and the sexual tabu which is imposed upon and readily accepted by women in general. Sex education as at present understood as the dissemination of knowledge of the " facts of life " is better than ignorance, but (at least so far as women are concerned) does not advance the situation very far. Too many people of both sexes assume that because the man achieves orgasm by friction of the penis in the vagina, the woman will or should achieve orgasm by friction of the vagina. This is not so, for at first such friction induces relatively little sensation. The homologue of the glans penis is the clitoris and it is here that the concentration of sensory nerve endings are located and it is by stimulation of this organ that the woman can achieve orgasm. Gradually the vaginal entrance and wall can be educated and conditioned so that coincident orgasm can be obtained. The author advises women to discover for themselves the correct rhythm of stimulation of the clitoris, and to achieve orgasm by digital manipulation and then to instruct their husbands to produce orgasm for them. At first coincidental orgasm cannot be expected, but later with conditioning of the vagina this may be achieved. If orgasm capacity is necessary for full happiness and fulfilment in married life for the woman (and there is much evidence that this is so) then we can recommend this book as a clear, concise and practical technical guide to this end.

**Conduct of Life Assurance Examinations.** By E. M. BROCKBANK, M.B.E., M.D., F.R.C.P. Second Edition. Pp. 176. London: H. K. Lewis & Co. Ltd. 1947. Price 12s. 6d.—The object of this book is to present in succinct form the general principles upon which Life Assurance Examinations should be carried out, and to provide a guide to the effect on expectation of life of the various disabilities which may be found. The tables showing the experience of American and British Companies in the various types of case are valuable. Here one may find, for instance, the effect upon longevity of obesity, of cardiac disorder and many other abnormalities. The author certainly has a very extensive knowledge of his subject, and his book provides in readily digested form a good deal of valuable information.

**Practical Food Inspection :** Vol. I. By C. R. A. MARTIN. Third Edition. Pp. viii., 316. Illustrated. London: H. K. Lewis & Co. Ltd. 1947. Price 18s.—The third edition of this already popular book will be welcomed by everyone concerned with the practical administration of food inspection. The author has confined the scope of the book to matters relating to cattle, sheep and pigs. The subjects dealt with embrace all phases of meat inspection such as physiology, comparative anatomy, ante- and post-mortem inspection, systems of meat inspection, slaughtering and dressing of animals, and the recognition of conditions physiological, pathological and parasitic. The book has been carefully indexed and incorporates a useful glossary of terms. The many illustrations have been carefully and meticulously drawn. The information contained in the book is up to date, accurate and of a very practical nature. It is pleasing for the simplicity of its language and the general presentation of material, features which will appeal to students in particular who so often read without comprehension. The author has purposely avoided superfluous detail and has concentrated on giving condensed, thorough, complete data on each subject dealt with.

**Nursing of Tuberculosis.** By O. V. BUXTON, S.R.N., and P. M. M. MACKAY, S.R.M.N. Pp. 124. Illustrated. Bristol: John Wright & Sons Ltd. 1947. Price 7s. 6d.—This book is written expressly for nurses engaged in the nursing of tuberculous patients. The title is perhaps unfortunate, as one expects from it actual nursing procedure in considerable detail on all points peculiar to tuberculosis nursing, whereas the book, in fact, is split into the orthodox sections, aetiology, bacillus, anatomy, symptoms and so on. Nursing procedure is, of course, included, but in somewhat scattered doses. A foreword by Dr. Trail states that "the book should be most useful to all Trained and Assistant Nurses who intend to take the certificate of the Tuberculosis Association." The diagrams are mediocre. Figure 1 with its captions does not conform to the written description in the text, and Figure 6 shows a rather unusual chest, with a pair of ribs missing and a diaphragm without visible associations with the thorax. Figure 8 is inaccurately labelled. Spontaneous pneumothorax receives no mention under the heading "Treatment of pulmonary symptoms"; it is the one real emergency in pulmonary tuberculosis. Later, in describing the treatment of the condition, the nurse, though expected to introduce a needle into the chest, gets no clear instructions into which side of the chest this should be done. It is stated that the early case is often difficult to diagnose except by radiography. Streptomycin is not mentioned, neither is calciferol nor the use of face masks in prevention. The use of pure lysol or pure carbolic in disinfection of flasks is dangerous to the nurse, and the recommended use of butter, eggs, milk and cream, though medically sound, savours of bygone days in this era of rationing. It would, however, be unfair to the authors to give only destructive criticism; the book contains a fund of valuable information, and a useful chapter on the psychological aspects is a very welcome addition.

